# The salamander blastema within the broader context of metazoan regeneration

**DOI:** 10.3389/fcell.2023.1206157

**Published:** 2023-08-11

**Authors:** Benjamin Tajer, Aaron M. Savage, Jessica L. Whited

**Affiliations:** Department of Stem Cell and Regenerative Biology, Harvard University, Cambridge, MA, United States

**Keywords:** blastema, limb, regeneration, salamander, evolution

## Abstract

Throughout the animal kingdom regenerative ability varies greatly from species to species, and even tissue to tissue within the same organism. The sheer diversity of structures and mechanisms renders a thorough comparison of molecular processes truly daunting. Are “blastemas” found in organisms as distantly related as planarians and axolotls derived from the same ancestral process, or did they arise convergently and independently? Is a mouse digit tip blastema orthologous to a salamander limb blastema? In other fields, the thorough characterization of a reference model has greatly facilitated these comparisons. For example, the amphibian Spemann-Mangold organizer has served as an amazingly useful comparative template within the field of developmental biology, allowing researchers to draw analogies between distantly related species, and developmental processes which are superficially quite different. The salamander limb blastema may serve as the best starting point for a comparative analysis of regeneration, as it has been characterized by over 200 years of research and is supported by a growing arsenal of molecular tools. The anatomical and evolutionary closeness of the salamander and human limb also add value from a translational and therapeutic standpoint. Tracing the evolutionary origins of the salamander blastema, and its relatedness to other regenerative processes throughout the animal kingdom, will both enhance our basic biological understanding of regeneration and inform our selection of regenerative model systems.

## Introduction

The salamander limb blastema is a transient, multipotent mass of mesenchymal cells that contributes to most major mesenchymal structures of the regenerated limb De Robertis. (2009) (Spallanzani, 1768; Bonnet, 1777) Arenas Gómez and Echeverri, 2021. The basic progression and dependencies of the blastema are well characterized (Thornton, 1938; Simon and Tanaka, 2013; Currie et al., 2016; Choi et al., 2017; Flowers et al., 2017; Haas and Whited, 2017; Gerber et al., 2018; Qin et al., 2021). The first steps of salamander limb regeneration appear quite similar to wound healing in humans: a blood clot quickly forms at the site of amputation, immune cells are recruited to the site of injury, and the adjacent epidermis quickly grows to cover the wound (Hay and Fischman, 1961; Endo et al., 2004; Ferris et al., 2010; Seifert et al., 2012). The next steps of salamander regeneration, however, diverge dramatically from mammalian wound healing. In mammals, myofibroblasts and keratinocytes enter the wound site and deposit fibrotic collagen, generating a scar (Jaźwińska and Sallin, 2016; Durant and Whited, 2021; Moretti et al., 2022)**.** In salamanders, the epidermis over the wound thickens into a specialized wound epidermis. Amputation triggers cellular proliferation inside stump tissues, but this proliferation is not restricted to the amputated limb and occurs within a subset of cells throughout the body (Johnson et al., 2018; Payzin-Dogru et al., 2023). The relationship between body-wide activation of prospective progenitor cells and local limb regeneration is not yet fully understood. Mesenchymal cells from the adjacent stump migrate to the site of injury, where they proliferate to form a visible bud. After an initial phase of outgrowth, blastema cells are specified and patterned to form structures in the regenerated limb. At this stage we consider the blastema complete, as the subsequent stages of regeneration primarily consist of the differentiation and growth of patterned structures (McCusker et al., 2015). Unsurprisingly, blastema growth is heavily dependent on cell division and is blocked by local irradiation (Rose et al., 1955; Thornton, 1958; Polezhaev, 1966). The formation and growth of the blastema also requires on innervation from the peripheral nervous system, with denervated limbs failing to form blastemas (Todd, 1823; Singer, 1946; Singer and Craven, 1948; Singer, 1952; Kumar and Brockes, 2012; Farkas and Monaghan, 2017).

The cellular origins and regenerative fate of blastema cells have been significant areas of research. In the early 20th century, pioneering analyses established that muscle, connective tissue, and bone are regenerated via the blastema, while the vasculature, nervous tissue, and epidermis reinvade or grow over the regenerating limb from outside the blastema (Towle, 1901; Weiss, 1925; Thornton, 1938; Goss, 1956). The exact nature and potency of the cells which contribute to the blastema is still debated to this day. In particular, the relative degree to which dedifferentiated mature cells and dedicated, resident progenitor/stem cells contribute to this process is unclear (Thornton, 1938; Lo et al., 1993; Morrison et al., 2006; 2010; Sandoval-Guzmán et al., 2014; Wang and Simon, 2016; Choi et al., 2017; Fei et al., 2017; Qin et al., 2021). Historically, it was assumed the blastema arose from dedifferentiated mature cells, and some recent studies support this (Thornton, 1938; Lo et al., 1993; Echeverri et al., 2001; Wang and Simon, 2016; Choi et al., 2017; Qin et al., 2021). In adult newts, even polynucleated muscle fibers can revert to a mononucleated state and contribute to the blastema (Wang et al., 2015; Wang and Simon, 2016). This phenomenon, however, is far from universal, with both larval newts and adult, neotenic axolotls repopulating muscle exclusively with progenitor satellite cells (Morrison et al., 2006; 2010; Sandoval-Guzmán et al., 2014; Tanaka et al., 2016; Fei et al., 2017). Recent lineage tracing studies suggest that the blastema is heterogenous, with several distinct subpopulations with independent origins and limited multipotency (Kragl et al., 2009; Currie et al., 2016; Choi et al., 2017; Flowers et al., 2017; Gerber et al., 2018). Some of these subpopulations may represent dedifferentiated mature cells, others undifferentiated progenitors, with most blastema cells deriving from mesenchymal and dermal fibroblasts, or perhaps fibroblast-like progenitor cells (Gerber et al., 2018; Leigh et al., 2018; Lin et al., 2021).

The regenerative blastema superficially resembles the developmental limb bud, and several studies have probed the function of developmental genes during regeneration. Important developmental signaling pathways, such as BMP, FGF, and Wnt appear to recapitulate some of their roles during regeneration: promoting tissue outgrowth, patterning morphological axes, and driving cellular differentiation (Ghosh et al., 2008; Guimond et al., 2010; Shimokawa et al., 2013; Makanae et al., 2014; Satoh et al., 2016; Wischin et al., 2017; Vieira et al., 2019). Hox genes, which specify proximal-distal identity in the limb bud, also recapitulate these roles in the late blastema: the regenerative expression pattern and tissue dependencies of proximal markers such as *Hoxa11* and *Meis1*, and distal markers such as *Hoxa13* mirror those in the limb bud (Gardiner et al., 1995; Fromental-Ramain et al., 1996; Torok et al., 1998; Post and Innis, 1999; Post et al., 2000; Carlson et al., 2001; Christen et al., 2003; Mercader et al., 2005; Woltering et al., 2019; Vincent et al., 2020; Takeuchi et al., 2022).

Salamanders have only recently become accessible to modern genetic analysis; in the last decade, experimental tools such as transgenesis, genome editing, targeted viral infection, single-cell RNA-seq, ATAC-seq, alongside genomic resources such as transcriptomes and full genome sequencing, have been developed, although many of these tools require significant optimization (Frahry et al., 2015; Keinath et al., 2015; Haas and Whited, 2017; Nowoshilow et al., 2018; Lertzman-Lepofsky et al., 2019; Smith et al., 2019; Schloissnig et al., 2021; Haley and Mueller, 2022). In addition to the aforementioned lineage tracing studies, this has enabled the large-scale transcriptional and proteomic profiling of gene expression in the blastema, revealing the upregulation of several interesting gene classes in blastema cells (Rao et al., 2009; 2014; Stewart et al., 2013; Bryant et al., 2017; Gerber et al., 2018; Leigh et al., 2018; Nowoshilow et al., 2018; Sibai et al., 2020 **).** These include several genes associated with pluripotency, oncogenesis, and mesenchymal identity such as *Myc*, *Atf3*, *Klf4*, *Klf2*, *Jun3*, *Egr1*, *Nr4a2*, *Fos*, *Eya1*, *Scx*, *Sox2*, *Foxd3*, and *Prrx1*, suggesting a link between regeneration and other proliferative processes (Stewart et al., 2013; Leigh et al., 2018). Genes associated with DNA damage repair, such as *Eya2, Rad51,* and *Mre11* are also upregulated in blastemas (Stewart et al., 2013; Sousounis et al., 2014; 2020; García-Lepe et al., 2021). Relatively few transcription factors are upregulated in the blastema, but several RNA binding proteins, such as *Cirbp*, *Fus*, *Roa1*, and *Hnrnpd* are strongly enriched, suggesting this may be a dominant mode of gene regulation during limb regeneration (Stewart et al., 2013; Bryant et al., 2017). Several metalloproteases (*Mmp1, Mmp2, Mmp3, Mmp8, Mmp9, MMmp10, Mmp12, Mmp13, Mmp12, Mmp19)* are also enriched, and the chemical inhibition of metalloproteases greatly disrupts regeneration, indicating ECM remodeling is also important (Yang et al., 1999; Vinarsky et al., 2005; Stevenson et al., 2006; Satoh et al., 2011; Stewart et al., 2013; Bryant et al., 2017).

In addition to these recognizable genes, many blastema-enriched transcripts are uncharacterized (Bryant et al., 2017). Several of these genes may be unique to salamanders, or may be ancestral to all tetrapods but lost in mammals (Dwaraka and Voss, 2021). Understanding the importance of these genes is critical to our understanding of blastema evolution, and the differential regenerative abilities of salamanders and mammals (Dwaraka and Voss, 2021). For example, the orphan gene *Prod1* is unique to salamanders, and is involved in proximo-distal patterning of blastema cells as well as digit outgrowth in newts (da Silva et al., 2002; Kumar et al., 2007a; 2015). Meanwhile, transcriptomic studies have revealed the upregulation of genes which are distantly related, highly divergent paralogs of mammalian genes such as *Cirbp* (also known as *Axrnbp*) and *Kazald2* in the blastema (Bryant et al., 2017). These data indicate that, in addition to genes shared with mammals, the salamander blastema may employ unique machinery either lost in mammals or gained in salamanders. While recent transcriptomics studies continue to expand our list of both characterized and uncharacterized blastema enriched candidates, only handful have been functionally interrogated (Sugiura et al., 2016; Fei et al., 2018; Sousounis et al., 2020). As the field examines more of these genes, our understanding of molecular blastema mechanisms will greatly improve.

Beyond the salamander limb field, the term “blastema” has been applied more generally to describe a large variety of structures, in many species, during the regeneration of several different organ systems (Reddien and Sánchez Alvarado, 2004; Bely and Nyberg, 2010; Fernando et al., 2011; Tanaka and Reddien, 2011; Bradshaw et al., 2015; Sallin et al., 2015; Zhao et al., 2016; Imperadore et al., 2017; 2022; Elchaninov et al., 2021; Bando et al., 2022; Vonk et al., 2022). These structures are generally superficially similar in that they consist of a mass of cells which must proliferate and repattern itself to regenerate large anatomical structures (Bely and Nyberg, 2010; Tanaka and Reddien, 2011; Zhao et al., 2016; Elchaninov et al., 2021)**.** While these phenomenological similarities allow us to draw general comparisons between a large variety of regenerative processes throughout the animal kingdom, the era of molecular biology empowers us to, and indeed demands that, we elucidate which of these processes are truly orthologous, which may simply meet the lower criterion of homologous, and which are only defensibly analogous. Determining which processes have a shared molecular basis, which processes have a common evolutionary origin, and which similarities are convergent, will have a profound effect on model selection, and eventual translation into patient therapies. How analogous is the regeneration of the mouse digit tip to that of a salamander limb? Are either of these processes truly related to the regeneration of the entire planarian body axis? In this review we position the salamander limb blastema as the archetypical blastema, owing to its clinically desirable ability to fully restore tetrapod forelimbs, and we explore how our current understanding of its molecular basis relates to analogous processes during development, wound healing, and during regeneration in other species.

Though many of the mechanistic details of salamander limb blastema formation, maintenance, and function remain undetermined, we can already identify enough key mechanistic and molecular features of the blastema to refine its definition beyond that of a simple proliferative outgrowth. As we further refine the interactions and circuitry of these elements, we will enhance our ability to make interspecies comparisons. As discussed later, regeneration likely originated alongside development at the very root of metazoan multicellularity (Bely and Nyberg, 2010); therefore, we should reasonably expect that the evolution of regenerative mechanisms will have much in common with the evolution of embryonic development where we see both conserved themes that span large segments of the animal kingdom, as well as lineage-specific derived modifications to this core program.

## An overview of metazoan regeneration: from salamander limbs to ctenophores

Salamanders and other amphibians diverged from other tetrapods around 330 million years ago, and they are notably the only members of this group which can fully regenerate their appendages as adults (Hedges et al., 1990; Zardoya and Meyer, 2001; Ruta et al., 2003; McCusker et al., 2015). Salamanders not only possess exceptional limb regeneration abilities, but can also regenerate several visceral organs such as the liver, heart, and gonad, and even brain structures (Detwiler, 1946; Erler et al., 2017; Dittrich et al., 2020; Lu et al., 2020; Ohashi et al., 2021; Lust et al., 2022; Wei et al., 2022). These abilities are not only lost or diminished in amniotes, but also in frogs, which share a more recent common ancestor with salamanders (Anderson et al., 2008). Frog tadpoles can regenerate limbs but gradually lose this ability over the course of metamorphosis (Suzuki et al., 2006; Simon and Tanaka, 2013; Mahapatra et al., 2023). Given this observation, it is tempting to assume that axolotl regenerative abilities arise from their neotenic lifestyle, but extensive regenerative capacities are found in all studied post-metamorphic salamanders, including both closely related ambystomids, as well as the most basal salamander groups Cryptobranchidae and Hynobiidae (Young et al., 1983; Griffin, 1995; Shen et al., 2013; Geng et al., 2015). Moreover, axolotls with induced metamorphosis can still regenerate, albeit at a slower pace and with reduced fidelity (Monaghan et al., 2014). Caecilians, the third extant group of amphibians, are poorly studied and do not have limbs, preventing a simple comparison with salamanders (Singarete et al., 2015).

While we typically consider amniotes to be poor regenerators, they can functionally recover from dramatic injuries to specific tissues, such as bone, and muscle, as well as specific visceral organs like the liver (Carlson, 2003; Ciciliot and Schiaffino, 2010; Abe et al., 2020; Delgado-Coello, 2021; Serowoky et al., 2022). Because humans possess these abilities, and because they are diminished in comparison to salamanders and fish, we tend not to classify them as “regeneration,” but many non-vertebrate animals lack these abilities (Bely and Nyberg, 2010; Elchaninov et al., 2021). Whether these regeneration events constitute a blastema is doubtful, however. Conversely, while most amniotes are unable to regenerate lost appendages as adults (Daponte et al., 2021), several mammals, including juvenile humans (and potentially older), and rodents throughout life, can regenerate digit tips, provided that the amputation leaves some of the most distal bone tissue, which goes through a blastema state (Illingworth, 1974; Neufeld and Zhao, 1995; Johnson and Lehoczky, 2022). Furthermore, many lizards are capable of regenerating tails when severed at a specific predetermined breaking point, though this regenerated tail lacks the complexity of the original (Gilbert et al., 2015). In both of these cases, it is unclear whether this regenerative ability represents a retained ancestral process, orthologous to salamander limb regeneration, or whether these abilities have been regained after being lost (Muneoka and Dawson, 2021). We can be more confident that the common ancestor of all tetrapods possessed salamander-like limb regeneration abilities. This is supported by the extensive appendage regeneration in lungfish, the closest extant relatives of tetrapods, and also by fossil evidence, which demonstrates that several ancient amphibian lineages, including lineages basal to the last common ancestor of modern amphibians and amniotes, could regenerate limbs (Fröbisch et al., 2014; Nogueira et al., 2016).

Beyond the Tetrapoda, extensive regenerative abilities are widespread amongst bony fish, which also employ blastemas during fin regeneration (Yoshinari and Kawakami, 2011; Darnet et al., 2019). It is likely that the common ancestor of all bony fish possessed axolotl-like regenerative abilities, and was capable of regenerating both endoskeletal elements and fin rays (Darnet et al., 2019). Supporting this, endoskeletal fin regeneration is seen in both basal sarcopterygians such as the lungfish, and basal actinopterygians such as the reed fish and paddlefish (Nogueira et al., 2016; Darnet et al., 2019). These ancestral regenerative abilities appear to be reduced in many lineages of teleost, including the widely studied zebrafish, which can only regenerate fin rays and dermal elements beyond larval stages (Darnet et al., 2019; Yoshida et al., 2020). Zebrafish, and many other teleosts, are still capable of extensive internal tissue regeneration in comparison to amniotes, and they have been prolific models for regenerative research, but the anatomical and regenerative differences between of limbs and fin rays limit the use of teleosts as a limb regeneration model (Gemberling et al., 2013; Pfefferli and Jaźwińska, 2015; Beffagna, 2019; Darnet et al., 2019; Marques et al., 2019).

Sharks and cartilaginous fish have only recently been demonstrated to exhibit enhanced muscle, cartilage, CNS and organ regeneration in comparison to amniotes (Lu et al., 2013; Alibardi, 2019; Borucinska et al., 2020; Marconi et al., 2020; Womersley et al., 2021; Alibardi, 2022a). As most of these studies are observational, the molecular basis of these processes remains unexamined, and it is unclear how closely they resemble salamander limb regeneration on the molecular level. Amongst the jawless fish, a large body of research has focused on the lamprey’s ability to regenerate spinal cord (Rasmussen and Sagasti, 2017; Hanslik et al., 2019), and recent research has investigated scar-free wound healing in lampreys (Li et al., 2023). Though adult lampreys seem incapable of appendage regeneration, larvae of at least three lamprey species are capable of tail regeneration, but the molecular basis of this process remains uninvestigated (Niazi, 1963; Bayramov et al., 2018). Regeneration in the hagfish appears to be unexplored. As these organisms sit at the base of the vertebrate tree, understanding their regenerative abilities and the underlying molecular mechanisms will be invaluable for our understanding of the ancestral vertebrate regenerative program.

Non-vertebrate deuterostomes generally possess extensive regenerative abilities (Ferrario et al., 2020). While the phylogeny at the base of the deuterostome clade is somewhat murky, urochordates, or tunicates, are commonly considered the closest relatives of the vertebrates; many possess truly extensive regenerative abilities, with some species capable of regenerating entire organ systems, large portions of the body, and even reproducing asexually (Rinkevich et al., 1995; 2007; Gordon et al., 2019; Ferrario et al., 2020). Though molecularly more distant than the tunicates, cephalochordates are more anatomically similar to vertebrates than adult tunicates, and their regenerative abilities would be more familiar to those who work on salamander and fish regeneration (Somorjai et al., 2012a; Ferrario et al., 2020). Lancelets can regenerate the post-anal tail through the formation of a blastema which appears to express some of the same markers as the salamander blastema, including Wnts and BMPs (Somorjai, 2017; Ferrario et al., 2020). Many echinoderms and hemichordates possess the ability to regenerate entire internal organ systems, large portions of the body, or in some cases even the entire body from an amputated appendage (Willey, 1900; Hyman, 1956; Ferrario et al., 2020). The regenerative abilities of echinoderms and basal chordates are well established, but the underlying molecular machinery is less so; understanding these mechanisms will be crucial to establishing whether there is any connection between the vertebrate blastema, and regenerative mechanisms throughout the broader animal kingdom.

Many protostomes also exhibit strong regenerative abilities. Protostomes are typically divided into two major groups, the Ecdysozoa, containing the arthropods, nematodes and their closest relatives, and Spiralia, which contains annelids, mollusks, and Platyhelminthes amongst others (Aguinaldo et al., 1997; Stechmann and Schlegel, 1999; Giribet, 2008)**.** Within Ecdysozoa, several arthropods have been documented to regenerate appendages (Bely and Nyberg, 2010; Suzuki et al., 2019; Brenneis et al., 2023). Arthropod limb regeneration appears to depend on migratory progenitor cells, and the formation of a proliferative blastema at the tip of the regenerating limb, though, interestingly, the outward morphological manifestation arthropod blastemas conforms to the molt cycle (Suzuki et al., 2019). A basal arthropod, *Pycnogonum littorale*, can also regenerate posterior structures including the gonad, suggesting the ancestral arthropod had similar abilities (Brenneis et al., 2023). Beyond Arthropoda, ecdysozoan regeneration is relatively unstudied, though nematodes are generally considered to be poor regenerators (Bely and Nyberg, 2010). Amongst Spiralia, several annelid and nemertean lineages can regenerate large portions of the anterior-posterior axis, while other lineages appear to have lost this ability completely (Bely et al., 2015). Mollusk regeneration is relatively unexplored, and research has primarily focused on the regeneration of neurons and neural structures in a limited number of groups (Moffett, 1995; 2000; Matsuo and Ito, 2011; Bely et al., 2015; Imperadore et al., 2017; De Sio and Imperadore, 2022). Cephalopods can regenerate limbs, but the underlying molecular biology of this process remains uninvestigated (Zullo et al., 2017; De Sio and Imperadore, 2022; Imperadore et al., 2022). When observed, regeneration in arthropods, annelids, and mollusks employs an epimorphic blastema, though the relatedness of these structures remains unresolved even within these clades.

Platyhelminthes, display varying degrees of regeneration (Bely et al., 2015). Planarians in particular have exceptional regenerative abilities and are by far the most studied and well understood invertebrate model for regeneration (Keller, 1894; Morgan, 1898; Baguñà et al., 1989; Reddien and Sánchez Alvarado, 2004; Reddien, 2018). Planarians are capable of regenerating the whole body from a small fragment utilizing body-wide stem cells, termed neoblasts (Keller, 1894; Morgan, 1898; Baguñà, 2012; Reddien, 2018). Neoblasts are the only dividing cells in the adult planarian and serve as the sole source of new material during tissue homeostasis (Baguñà et al., 1989; Newmark and Sánchez Alvarado, 2000; Eisenhoffer et al., 2008; van Wolfswinkel et al., 2014; Reddien, 2018). Planarian neoblasts are heterogenous, with most neoblasts exhibiting a limited degree of multipotency (Reddien, 2013). Some neoblasts, however, remain totipotent and can replenish all other neoblast subpopulations (Wagner et al., 2011; Reddien, 2018; Ge et al., 2022). During planarian regeneration neoblast cells throughout the body proliferate and migrate to the site of injury where they initiate a second wave of proliferation, creating an epimorphic blastema outgrowth (Baguñà, 1976; Saló and Baguñà, 1984; Wenemoser and Reddien, 2010). Once the blastema is established, regenerated tissue is patterned by Wnt and Bmp, which also control axial polarity and patterning during embryogenesis (Molina et al., 2007; Petersen and Reddien, 2008; Reddien, 2018). Planarians express Wnt and Bmp gradients throughout their lives, and rapidly readjust them in response to injury; with muscle cells secreting these ligands (Witchley et al., 2013; Reddien, 2018). This sequence of body wide proliferation followed by migration and subsequent blastema outgrowth bears superficial resemblance to salamander blastema formation, which also redeploys the developmental signaling molecules Wnt and BMP, perhaps hinting at a common ancestral regenerative program (McCusker et al., 2015; Johnson et al., 2018; Srivastava, 2021).

From an evolutionary perspective, perhaps the most striking aspect of planarian regeneration is its strong similarity to acoel regeneration (Srivastava et al., 2014; Gehrke and Srivastava, 2016; Raz et al., 2017; Srivastava, 2022). Though morphologically similar to planarians, recent phylogenetic analyses place acoels either amongst the deuterostomes, or at the base of bilateria; in either scenario acoels are only distantly related to planarians (Srivastava, 2022). Like planarians, acoels possess both totipotent and heterogeneous neoblast-like stem cells that provide all new tissue during both homeostasis and regeneration (Srivastava, 2022). Wnt and Bmp are also expressed in acoel muscle cells, in a graded fashion, along major body axes, and control positional information (Raz et al., 2017; Srivastava, 2022). The similarities between regeneration in these two distantly-related groups strongly hint at a shared regenerative mechanism in the last common ancestor of all bilateral animals; this program may be antecedent to the salamander blastema, with common features such as the proliferation and migration of progenitor cells being conserved elements of this program (Srivastava, 2022).

Beyond Bilateria, strong regenerative abilities are seen in cnidarians, sponges, and placozoans (Holstein et al., 2003; DuBuc et al., 2014; Ereskovsky et al., 2021; Fujita et al., 2021; Osigus et al., 2022; Romanova et al., 2022). In these organisms, the lines between regeneration, development, and asexual reproduction are more blurred than in most studied bilateral species (Bely and Nyberg, 2010; Slack, 2017; Martinez et al., 2022; Rinkevich et al., 2022). Many cnidarians are capable of regenerating large portions of their bodies as well asexual reproduction (Bely and Nyberg, 2010; Slack, 2017; Martinez et al., 2022; Rinkevich et al., 2022). Cnidarians also possess stem cells which may be functionally similar to planarian neoblasts, such as i-cells in hydrozoans, and amoebocytes in other groups, but the majority of regenerated tissue typically comes from “mature” epithelial cells which are also somewhat multipotent and proliferative (Gold and Jacobs, 2013; Martinez et al., 2022; Rinkevich et al., 2022). Sponges also have great regenerative abilities, and as with cnidarians, “mature” sponge cells can often proliferate and transdifferentiate (Ereskovsky et al., 2021). Generally within these organisms, most cells are proliferative and retain some degree of multipotency, making stem cells somewhat hard to define (Rinkevich et al., 2022). Though the cellular basis for regeneration in these basal animal groups is quite different from that employed in the salamander blastema, shared regulatory features may still govern regeneration in both contexts (Arendt et al., 2016; Srivastava, 2021; Rinkevich et al., 2022).

Ctenophores exhibit variable regenerative abilities, ranging from whole-body regeneration in some species to the complete absence of adult regeneration in others (Martindale, 2016; Ramon-Mateu et al., 2019; Edgar et al., 2021). Ctenophores may be the most basal metazoan group and are likely to inform our understanding of regeneration in the last common ancestor of all animals (Martindale, 2016; Edgar et al., 2021). Ctenophores appear to replace lost tissues through the proliferation of differentiated cells of the same type, yet the nuances of this process remain unresolved and the extensive regeneration abilities of several species suggest that some transdifferentiation probably occurs (Edgar et al., 2021). As with sponges and cnidarians, it is unclear if traditional distinctions between stem cells and differentiated cells truly apply within this group (Edgar et al., 2021; Rinkevich et al., 2022). Moreover, mesogleal cells appear to migrate to the wound site in ctenophores, superficially resembling the migration of mesenchymal or ameboid cells seen during regeneration in other groups, and, possibly the formation of the salamander blastema, but the role of these cells remains unclear (Edgar et al., 2021). Intriguingly, ctenophores lack FGF, and have uniquely evolved and elaborated gene families for other metazoan signaling pathways, such as TGF-β and Wnt (Pang et al., 2010; 2011; Moroz et al., 2014; Edgar et al., 2021). Accordingly, any conserved regulatory mechanisms between ctenophores and other animals would be extremely fundamental, preceding the subsequent diversification of these major gene families. Currently, the evolutionary distance of ctenophores and lack of molecular data places such comparisons beyond our reach. Ultimately our understanding of the evolution of animal regeneration, and the limb blastema more specifically, will require a more thorough molecular and mechanistic interrogation of many species on all major branches of the animal tree.

## Using gene regulatory network analyses and tissue dependencies to frame blastema evolution

It is important to acknowledge the significant challenges we face when comparing sophisticated biological processes such as regeneration across vast evolutionary distances. Processes like regeneration evolve at several levels of abstraction in comparison to the evolution of species or of gene families (Liberles and Dittmar, 2008; Arendt et al., 2016; Elchaninov et al., 2021; Srivastava, 2021). Processes are generally considered to be homologous when they derive from a common ancestral process, but the establishment of this common ancestry is fraught. Conservation of molecular components is a good starting point, but the replacement of individual components in different lineages can mask a shared origin, while the independent employment of the same genes by convergently evolved processes misleadingly suggest a shared origin (Striedter and Northcutt, 1991; Arendt et al., 2016; Elchaninov et al., 2021; Srivastava, 2021).

Considering these complications, gene regulatory networks (GRNs) are a promising tool for establishing homology. While individual GRN components, such as genes and genomic regulatory elements, may be lost or replaced over evolutionary time, cumulatively these circuits should remain relatively intact (Davidson et al., 2002; Davidson, 2006; Srivastava, 2021). Likewise, independently evolved processes may convergently employ similar genes, but they are unlikely to incorporate the same combinations of these elements or the same regulatory interactions (Srivastava, 2021). Accordingly, truly homologous GRNs should share several significant components, while convergently evolved GRNs may share a few genes but should generally be quite different (Srivastava, 2021). Two potential GRNs, an injury-induced Erk-Wnt circuit, and a Germline Multipotency Program (GMP), appear to be widely conserved in animal regeneration (Srivastava, 2021). Unfortunately, the identification and validation of such gene regulatory networks in regeneration remains difficult, since cross-phyla molecular data across is lacking (Rinkevich et al., 2022). Moreover, the establishment of *bone fide* GRNs requires functional data, a well-characterized genomic sequence, and ideally epigenetic information in addition to gene expression data (Davidson et al., 2002; Davidson, 2006; Srivastava, 2021).

GRNs can also be used to trace the evolution of cell type. Specific GRNs, recently termed Core Regulatory Complexes (CoRCs) enforce the identity of distinct cell types (Arendt et al., 2016). Over the course of evolution, CoRCs diverge from each other, either through the duplication of genetic components or through the integration of new regulatory machinery (Arendt et al., 2016). When the regulatory basis of two CoRCs is sufficiently different, the selective pressure maintaining these “sister” cell type identities becomes unlinked, and the new cell types can be considered distinct (Arendt et al., 2016). This concept is notable, because it allows researchers to trace the evolution of cell types to characteristic regulatory modules which can be investigated independently of morphology, host species, and developmental origin (Arendt et al., 2016). The characterization and comparison of relevant blastema CoRCs throughout the animal kingdom should allow us to tell the degree to which these cells, and by extension these processes, are related.

As previously mentioned, our ability to compare regeneration across animal phyla is complicated by a lack of molecular data from several major groups, with many taxa represented by only a handful of species, or none at all (Rinkevich et al., 2022). Even the well-established axolotl suffers from poor genome annotation compared to more established genetic models such as mouse and zebrafish (Frahry et al., 2015; Keinath et al., 2015; Nowoshilow et al., 2018; Lertzman-Lepofsky et al., 2019; Smith et al., 2019; Dwaraka and Voss, 2021; Schloissnig et al., 2021; Haley and Mueller, 2022). That said, over the last 10 years, transcriptomic and genomic studies have already greatly enhanced our understanding of several phyla, especially cnidarians, and acoels, and we are likely to make rapid progress in the coming years (Ferrario et al., 2020; Rinkevich et al., 2022; Srivastava, 2022). This influx of molecular data does come with some caveats. Sequencing efforts are biased towards emphasizing commonalities and understating differences; conserved genes are easier to annotate in models with rudimentary and/or unreliable annotation, and naturally will draw more attention when they show up in gene expression lists (Ferrario et al., 2020; Rinkevich et al., 2022). Generating hypotheses for uncharacterized genes is more challenging than for characterized genes, though these may have significant biological importance and even clinical relevance (Ferrario et al., 2020; Rinkevich et al., 2022).

Given these challenges, one might wonder, why even try to trace the evolution and interrelatedness of metazoan regenerative mechanisms? One motivation is pure, basic-biological curiosity. Regeneration is widespread throughout the animal kingdom, suggesting an origin at the very base of the animal tree, and appears to be connected to other processes like development and pluripotency, which lie at the very heart of animal multicellularity itself (Bely and Nyberg, 2010; Slack, 2017). Tracing the evolution of regeneration is likely to enhance our understanding of these processes. Regenerative abilities have also been reduced in many animal taxa and may have been secondarily enhanced or reacquired within subgroups within these phyla; a general understanding of regenerative evolution will inform our understanding of the selective pressures that act on regeneration and illuminate physiological and developmental tradeoffs involved with retaining and losing regenerative abilities (Bely and Nyberg, 2010; Elchaninov et al., 2021). This motivation has already inspired several reviews (Sánchez Alvarado, 2000; Bely and Nyberg, 2010; Elchaninov et al., 2021).

There is also a practical motivation. As mentioned previously, understanding homology between regenerative processes will aid in our selection of model systems; this is particularly important from a translational perspective. As salamanders are the only tetrapods capable of complete limb regeneration, the salamander limb blastema represents an aspirational goal for the field of regenerative medicine (Fior, 2014). If we can understand which key features of the salamander blastema are shared by other systems, we may be able to study these processes in organisms with easier husbandry and friendlier genetics, such as fish and planarians. Likewise, we will be able to assess the therapeutic validity of more closely related models of regeneration, such as the mouse digit tip and the lizard tail; if these processes are secondarily derived, they may not be as informative towards the goal of whole appendage regeneration. It is worth emphasizing that such comparisons are capable of bearing fruit. As previously mentioned, developmental biologists have identified a conserved Spemann-Mangold organizer program demonstrating that we can establish evolutionary continuity, homology, and convergence between complex processes (De Robertis, 2009). The field stands at an exciting juncture, armed with new heuristics for understanding the evolution of GRNs and cell types, and as we acquire molecular and functional data from an expanding menagerie of species across the evolutionary tree we will be able to make these comparisons with a much greater degree of authority and specificity in the coming years.

For comparative purposes we will focus on specific functional features and molecular aspects of the salamander blastema. The salamander limb blastema can be thought of as having several key ingredients: a protective wound epidermis which forms over the nascent blastema and promotes its growth, a population of mesenchymal cells which is the substrate of blastema formation, a neural contribution, and molecular signals that guide and organize cell behaviors globally and locally (Simon and Tanaka, 2013; McCusker et al., 2015; Payzin-Dogru and Whited, 2018). In this review, we will give particular focus to the conservation of nerve dependence, the mesenchymal/stem cells which contribute to the blastema, the progenitor status of these cells, and the pluripotency associated factors expressed and GRNs within these blastema cells. Though we are primarily interested in appendage regeneration, we will also integrate insights from whole-body regeneration, visceral organ regeneration, and stem cell biology more generally when relevant.

### Is nerve dependence an ancestral feature of the vertebrate blastema?

The importance of neurons and supporting tissues/cells (such as Schwann cells) appears to be widespread in vertebrate regeneration: in addition to the salamander blastema, it is a feature of mouse digit tip regeneration, the regeneration of various tissues throughout the mouse body, such as the heart, the regeneration of the lizard tail, and the regeneration of the zebrafish fin (Kumar and Brockes, 2012; Bely, 2014; Simões et al., 2014; Pirotte et al., 2016; Farkas and Monaghan, 2017; Storer and Miller, 2020)**.** Nerves appear to have important functional roles in the regeneration of several invertebrates (Kumar and Brockes, 2012; Pietak et al., 2019; Suzuki et al., 2019). In some annelids, echinoderms, and cephalopods, presumptive blastema cells appear to migrate along nerves towards the site of injury (Ferrario et al., 2020; Kostyuchenko and Kozin, 2021; Imperadore et al., 2022). In planarians the polarity of the residual nervous system partially informs the morphology of the regenerating body axis (Kumar and Brockes, 2012; Pietak et al., 2019). In *Drosophila*, innervation supports various stem cell niches, similar to its roles in mammalian tissue regeneration (Brückner, 2011; Makhijani et al., 2011; Kumar and Brockes, 2012)**.** Even in cnidarians, where neurons are not obligatorily required for regeneration, they promote regeneration (Miljkovic-Licina et al., 2007; Kumar and Brockes, 2012). The widespread involvement of nerves in regeneration hints at deep evolutionary origins for this feature of the salamander limb blastema (Bely and Nyberg, 2010; Kumar and Brockes, 2012). This feature is also of particular interest because it marks a major difference between limb development and regeneration, as the initial limb bud forms prior to innervation (Farkas and Monaghan, 2017).

Much interest has focused on the molecular factors underlying the relationship between nerves and the salamander blastema. The neurotropic hypothesis postulates that neurons secrete factors which support and maintain the salamander limb blastema, and stands as the dominant paradigm within the field (Singer, 1964; 1978; Pirotte et al., 2016; Farkas and Monaghan, 2017). Researchers have identified FGFs, BMPs, Insulin, Transferrin, Substance P, NGF, Newt Anterior gradient, Neuregulin-1, Oncostatin M, and PDGF-AA as potential candidates for such neurotropic factors, as they are secreted by neurons, reduced in the case of denervation, and promote the proliferation of blastema cells (Vethamany-Globus and Liversage, 1973; 1973; Globus, 1978; Albert et al., 1987; Anand et al., 1987; Globus et al., 1991; Kiffmeyer et al., 1991; Mescher et al., 1997; Wang et al., 2000; Christensen et al., 2001; Kumar et al., 2007b; Makanae et al., 2013; Farkas et al., 2016; Grassme et al., 2016; Johnston et al., 2016; Pirotte et al., 2016; Satoh et al., 2016; Farkas and Monaghan, 2017). BMPs and FGFs are known to play a variety of roles in regeneration and development throughout the animal kingdom, although the presence of these factors alone does not necessarily involve communication between neurons and the blastema (Molina et al., 2007; Reddien et al., 2007; Maddaluno et al., 2017; Slack, 2017). NGF has been observed in echinoderm and annelid regeneration (Patruno et al., 2001; Thorndyke and Carnevali, 2001; Kostyuchenko and Kozin, 2021), while planarian regeneration appears to employ a different set of neuronal factors (Reddien et al., 2005a; Pirotte et al., 2016). The presence of other axolotl neurotropic candidates has not been noted in invertebrates; this could be because, as with planarians, a different set of factors is involved in these systems. If this is the overall trend it could suggest that nerve dependence evolved convergently multiple times in different lineages. Ultimately the lack of an established mechanism for nervous system contribution during salamander regeneration, and a lack of molecular studies that characterize nervous system involvement in invertebrate appendage regeneration limit our ability to directly compare nervous dependence across these systems (Farkas and Monaghan, 2017; Kostyuchenko and Kozin, 2021). Furthermore, it should be noted that “nerve” is a squishy term that encompasses both neurons and accessory cell types, such as Schwann cells and others, which may very likely play important roles but whose contributions have not been cleanly parsed out.

If nerve dependence is an ancestral feature of regeneration, the evolutionary history of this feature should be intertwined with the evolution of the nervous system more generally. All four basal animal clades exhibit strong regenerative abilities, yet sponges and placozoans completely lack neurons, and denervated cnidarians are still capable of regeneration (Miljkovic-Licina et al., 2007; Kumar and Brockes, 2012; Edgar et al., 2021; Ereskovsky et al., 2021; Osigus et al., 2022). This may mean nerve dependence evolved at the base of Bilateria, or that this feature was independently acquired multiple times in unrelated bilaterian lineages. Alternatively, the molecular mechanism that underlies nerve dependence may have preceded the development of a distinct neural cell type and has been acquired by non-neural cells in these lineages. The recent, controversial placement of ctenophores at the base of the metazoan tree challenges our traditional understanding of nervous evolution, suggesting that the nervous system was either an ancestral trait of all metazoans that was secondarily lost in sponges and placozoans, or that neurons evolved independently in ctenophores and eumetazoans (Cnidaria and Bilateria) (Moroz et al., 2014; Colgren and Burkhardt, 2022). If the molecular machinery that underpins nerve dependence is truly ancient, we may be able to use the manifestation of this mechanism in basal animal lineages to discern between these two hypotheses.

Though nerve dependence may be widespread, there are notable examples of nerve independence in systems where nerves are typically required (Filoni et al., 1995; 1999; Suzuki et al., 2005; Farkas and Monaghan, 2017). Denervated salamanders can be produced through the removal of the neural tube during embryogenesis, and they can be maintained through parabiosis with an otherwise unmanipulated host with an intact nervous system (Yntema, 1959). Surprisingly, these animals regenerate amputated limbs normally without any innervation (Wallace, 1980; Filoni et al., 1995; 1999; Tassava and Olsen-Winner, 2003; Suzuki et al., 2005; Satoh et al., 2011), suggesting nerve dependence only occurs after initial innervation. Similarly, the *Xenopus* tadpole is capable of forming a nerve-independent blastema, but *Xenopus* blastema formation becomes increasingly nerve dependent over the course of development (Filoni et al., 1995; 1999; Suzuki et al., 2005; Farkas and Monaghan, 2017).

These examples strongly suggest that even in these “nerve dependent” systems, regeneration can still occur in the absence of nerves. Resolving the mechanistic differences between nerve-dependent regeneration and nerve-independent regeneration in these amphibian models will be key to understanding the evolution of nerve dependence, at least within the vertebrate lineage. Is the neural program delegated to a different cell type in the nerve-independent examples, or is a different mechanism employed entirely? An interesting hypothesis is that after their initial development, regenerating limbs become “addicted” to neurogenic factors (Kumar et al., 2011). Perhaps during vertebrate, or even bilaterian, evolution, regeneration transitioned from a nerve-independent to a nerve-dependent process. Likewise, the involvement of nerves in salamander limb regeneration may illuminate the loss of limb regeneration in amniotes, where limbs develop in a less mature tissue environment than in salamanders (Borgens et al., 1977; Borgens, 1984; Borgens et al., 1987; Alibardi, 2022a).

While much research has focused on the neurotropic hypothesis, less attention has focused on alternative mechanisms for nervous system involvement (Singer, 1978; Farkas and Monaghan, 2017). Recent findings suggest that innervation is required for the body-wide proliferation of stem cells after injury in the axolotl (Payzin-Dogru et al., 2023). Others have proposed that the nervous system encodes and directs the ultimate target morphology of the regenerated appendage, reflecting the observed impact ectopic innervation on blastema morphology (Stocum, 1991; Levin, 2012). Intriguingly, cancer tumors also depend on innervation, hinting at shared cellular mechanisms between regeneration and cancer (Levin, 2012; Boilly et al., 2017; Wong and Whited, 2020). Moreover, adrenergic signaling has been implicated in both metastasis and in the nerve-dependent, body-wide cell cycle activation during axolotl regeneration (Nagaraja et al., 2016; Payzin-Dogru et al., 2023). When considered alongside the superficial observations that both tumors and blastemas employ the proliferation of a dedifferentiated cell mass, these findings support the hypothesis that cancer tumors redeploy regenerative machinery (Levin, 2012; Wong and Whited, 2020). From this vantage point, our understanding of the limb blastema may yield therapeutic insights into novel cancer treatments. Cumulatively these findings suggest that nervous contributions to the blastema are more complex and varied than is commonly appreciated; understanding these uncharacterized functions will likely facilitate future comparative studies.

### Are mesenchymal cells a conserved feature of blastema formation?

Understanding and comparing the cell types which contribute to the blastema in different animal lineages is key to tracing the homology of regenerative mechanisms. In the salamander, the bulk of the cells that contribute to the blastema are of fibroblast origin (Muneoka et al., 1985; 1986; Gerber et al., 2018; Leigh et al., 2018), and there is evidence to suggest these cells provide the bulk of blastema material in other vertebrates, including *Xenopus*, fish, mice and lizards (Johnson and Bennett, 1998; Sehring and Weidinger, 2020; Storer and Miller, 2020; Lin et al., 2021; Alibardi, 2022b; Hu et al., 2022). Though fibroblasts have been conventionally considered a mature cell type, specialized in the maintenance of intracellular matrix, increasing evidence supports the idea that at least some fibroblasts serve as dedicated progenitor cells during tissue homeostasis (LeBleu and Neilson, 2020; Plikus et al., 2021). Recent studies leveraging single-cell RNA-seq and *in vivo* fate mapping have revealed considerable heterogeneity in fibroblast subtypes and their differing behaviors following injury (Jiang et al., 2018; Leigh et al., 2018; Jiang and Rinkevich, 2021; Sinha et al., 2022; Talbott et al., 2022). Fibroblast migration, accumulation, and proliferation are also features of scar formation in non-regenerative vertebrates (Jaźwińska and Sallin, 2016; Jiang et al., 2018; Jiang and Rinkevich, 2021; Moretti et al., 2022; Talbott et al., 2022), hinting that in some ways, a scar could be considered a vestigial or modified blastema.

Fibroblast-like cells have been observed in both mollusk and echinoderm appendage regeneration (Ben Khadra et al., 2015; Furukawa et al., 2021), but the direct contribution of these cells to regenerating tissue remains unexamined. Indeed, there seems to be relatively little comparative analysis of the fibroblast cell type across major animal taxa. Planarians, acoels, cnidarians, and annelids utilize multipotent progenitors termed neoblasts, amoebocytes, or i-cells during regeneration (Gold and Jacobs, 2013; Raz et al., 2017; Reddien, 2018, 201; Kostyuchenko and Kozin, 2021; Rinkevich et al., 2022). These cells are superficially similar to each other and to the fibroblasts utilized in the salamander blastema owing to their interstitial residence, migratory behavior, proliferative potential, and multipotency, but the homology of these cell types between major taxa is unconfirmed and is even contentious between different lineages within Cnidaria and Annelida (Gold and Jacobs, 2013; Reddien, 2018; Kostyuchenko and Kozin, 2021; Srivastava, 2022). Moreover, while cnidarian i-cells and amoebocytes most closely resemble planarian neoblasts, they do not contribute to the majority of regenerated tissue in cnidarians, instead this material is provided by transdifferentiating epithelial cells, which appear to have a greater degree of multipotency than similar cells in other animal groups (Gold and Jacobs, 2013; Rinkevich et al., 2022).

Migratory ameboid cells also appear to play a major role in regeneration throughout the animal kingdom. In axolotls, fish, and the mouse digit tip, macrophages and other myeloid cells migrate to the injury site, where they are not only involved in stereotypical macrophage roles, such as clearing infectious pathogens and removing cellular debris, but they appear to be necessary for the promotion and maintenance of the subsequent blastema (Fernando et al., 2011; Godwin et al., 2013; Morales and Allende, 2019; Bohaud et al., 2021). Recent experiments have shown that macrophages are obligate required for blastema formation and outgrowth in the axolotl limb, zebrafish fin, and mouse digit tip (Fernando et al., 2011; Godwin et al., 2013; Morales and Allende, 2019; Bohaud et al., 2021). Notably in macrophage-depleted axolotls, wounds can still heal, but regeneration is disrupted, suggesting a profound role for macrophages in the establishment and maintenance of the blastema itself, independent of their canonical immune functions (Godwin et al., 2013). Circulating ameboid cells can also be found in the arthropod, annelid, echinoderm, ascidian, and mollusk blastemas (Pinsino et al., 2007; Hernroth et al., 2010; Rinkevich et al., 2010; Gold and Jacobs, 2013; Imperadore et al., 2017; 2022; Suzuki et al., 2019; Kostyuchenko and Kozin, 2021). These cells go by many different names: plasmocytes, hemocytes, Coelomocytes, and amoebocytes. During regeneration, they appear to take on many different roles, including phagocytosis, clot formation, and even direct cellular contribution to the blastema itself (Pinsino et al., 2007; Hernroth et al., 2010; Gold and Jacobs, 2013; Imperadore et al., 2017; 2022; Suzuki et al., 2019; Kostyuchenko and Kozin, 2021). At certain stages of regeneration these cells make up the major component of the blastema in some echinoderms and cephalopods, but their ultimate contribution remains unclear in the absence of lineage tracing (Pinsino et al., 2007; Imperadore et al., 2017; 2022).

On the most superficial level, there is a common theme by which interstitially resident, non-epithelial cells migrate to and accumulate at the blastema. Their superficial resemblance to basal stem cells such as sponge archaeocytes or cnidarian amoebocytes, and migratory resemblance to blastema fibroblasts present a tantalizing hypothesis: that these cells descend from a shared ancestral regenerative cell type, the roles of which have been sub functionalized to different sister cell types in different animal lineages. At present such a hypothesis is extremely speculative, but the increased cellular characterization of invertebrate models coupled with the CoRC frame work may allow us to test such hypotheses in the near future (Arendt et al., 2016).

### When did the use of dedifferentiation and progenitor cells evolve in the salamander blastema and other regenerative systems?

The relative contribution of dedicated progenitor stem cells and dedifferentiated mature cells has been a major area of focus in several regenerative systems (Bely and Nyberg, 2010; Rinkevich et al., 2022). In this regard there is considerable variation across the animal kingdom, and the means by which we distinguish between these processes depends on how we define “mature” and “progenitor” cells (Rinkevich et al., 2022). Some systems, such as planarians and acoels, have well defined stem cells: neoblasts are specified during embryonic development, retain an unspecialized morphology, and are the only proliferative cells in the adult during both homeostasis and regeneration (Wenemoser and Reddien, 2010; Raz et al., 2017; Kimura et al., 2022; Hulett et al., 2023). Cnidarians and sponges on the other hand, have multiple proliferative cell types with varying degrees of multipotency (Gold and Jacobs, 2013; Edgar et al., 2021; Ereskovsky et al., 2021; Rinkevich et al., 2022). Though these organisms also possess apparent adult stem cells, such as i-cells, amoebocytes, and archaeocytes, which retain an unspecialized morphology and have a greater degree of multipotency, the bulk of regenerated material in these species comes from the proliferation of specialized epithelial cells (Gold and Jacobs, 2013; Ereskovsky et al., 2021). The proliferation and outgrowth of mature tissues is also a major component of regeneration in ctenophores, echinoderms, tunicates, and several annelids (Ferrario et al., 2020; Edgar et al., 2021; Kostyuchenko and Kozin, 2021). Several echinoderms and tunicates also employ dedifferentiation, where morphologically mature, specialized cells revert to a less specific, often migratory morphology, before redifferentiating into new mature cell types (Rinkevich et al., 2010; Voskoboynik and Weissman, 2015; Ferrario et al., 2020). These strategies are not mutually exclusive and are combined in many systems, including cnidarians, tunicates, echinoderms, annelids, and most notably for this review, salamanders (Rinkevich et al., 2010; 2022; McCusker et al., 2015; Voskoboynik and Weissman, 2015; Ferrario et al., 2020). In the salamander limb, several tissues, including the skin, nerves and vasculature, are largely produced through the proliferation of resident, mature tissues; the blastema bud itself appears to consist of heterogenous progenitor cells some of which may originate from dedifferentiated mature cells (Kragl et al., 2009; McCusker et al., 2015; Tanaka et al., 2016; Choi et al., 2017; Leigh et al., 2018; Dwaraka and Voss, 2021)**.**

It was long thought that all cells within the axolotl blastema arose through dedifferentiation and constituted a single multipotent blastema cell type, capable of regenerating all mesenchymal tissues within the axolotl limb (Thornton, 1938; Smith and Wolpert, 1975). Recent studies have challenged this view, revealing that the blastema contains many heterogenous cell populations with limited multipotency (Kragl et al., 2009; Choi et al., 2017; Flowers et al., 2017; Gerber et al., 2018; Leigh et al., 2018; Currie et al., 2019). Moreover, while some transdifferentiation may occur during limb regeneration, most differentiated cells appear to be derived from progenitors of the same, or closely-related, lineages within the original limb (Kragl et al., 2009; McCusker et al., 2016; Choi et al., 2017; Leigh et al., 2018). This aspect of salamander regeneration resembles planarian and acoel regeneration, which also employ heterogenous migratory progenitor cells, although unlike axolotls, these groups possess a truly pluripotent adult stem cell population capable of restoring all progenitor classes (Wagner et al., 2011; Reddien, 2013; Gehrke and Srivastava, 2016; Ge et al., 2022; Hulett et al., 2023).

Given the heterogeneity and potential multipotency and proliferative abilities of the many blastema fibroblast populations, whether they are truly progenitors or dedifferentiated cells remains unclear (LeBleu and Neilson, 2020; Plikus et al., 2021). One clear example of dedifferentiation, is the dedifferentiation of polynucleated muscle fibers during post-metamorphic newt limb regeneration (Sandoval-Guzmán et al., 2014; Tanaka et al., 2016; Dwaraka and Voss, 2021). If this trait is limited to salamanders, it would suggest that this is a derived mechanism, but the absence of non-salamander examples may simply reflect a lack of studies which specifically interrogate muscle dedifferentiation outside the salamander clade. The distribution of this mechanism across salamander taxa also remains unclear; while it is employed by post metamorphic newts (Salamandra clade), it is not used in axolotls (Ambystoma clade) with forced metamorphosis (Sandoval-Guzmán et al., 2014; Dwaraka and Voss, 2021). The sheer cytological complexity and sophistication of this process suggest it likely evolved over a long period of time, and the observation of superficially similar phenomena in echinoderms and annelids, may hint at a more ancient origin (Sandoval-Guzmán et al., 2014; Tanaka et al., 2016; Ferrario et al., 2020; Kostyuchenko and Kozin, 2021). Ultimately, investigations in less characterized salamander families will illuminate the evolutionary provenance of this mechanism (Dwaraka and Voss, 2021).

Variations in the regenerative deployment of progenitor cells or dedifferentiation across the animal kingdom, generally reflect the underlying stem cell logic in those organisms (Rinkevich et al., 2022). This logic exists on a continuum: on one extreme, as seen in acoels and planarians, dedicated, undifferentiated stem cells are the only proliferative cell class and provide all material during regeneration; on the other extreme, as in many sponges and cnidarians, most mature cell types are proliferative and retain some degree of multipotency (Gold and Jacobs, 2013; Reddien, 2018; Ereskovsky et al., 2021; Srivastava, 2022). Basal metazoans generally sit towards the latter end of this continuum, while bilateral lineages sit at different positions, with vertebrates and arthropods relying more heavily on dedicated stem cells, some echinoderms and tunicates relying more on mature cells, and many annelids sitting somewhere in the middle (Ferrario et al., 2020; Kostyuchenko and Kozin, 2021; Rinkevich et al., 2022). From this distribution we can infer that ancestral metazoans most likely possessed multiple proliferative mature cell types, with the increasing use of dedicated stem cells emerging later (Rinkevich et al., 2022). It is less obvious whether this transition occurred in the basal bilaterian, or if dedicated stem cells convergently evolved in different bilateral lineages. Supporting a common origin, there are remarkable similarities between planarian and acoel neoblasts, despite their vast evolutionary distance, suggesting that the ancestral bilaterian regenerated through a similar, stem-cell-exclusive mechanism (Gehrke and Srivastava, 2016; Raz et al., 2017). If this is the case, several bilaterian lineages, in particular several tunicates and echinoderms, which utilize both mature cell proliferation and dedifferentiation, would have re-evolved these mechanisms (Auger et al., 2010; Jeffery, 2015; Ferrario et al., 2020). Supporting this in the most basal echinoderm group, the crinoids, regeneration appears to employ dedicated stem cells, in a manner which more closely resembles vertebrate, planarian, and acoel regeneration (Candia Carnevali and Bonasoro, 2001; Ferrario et al., 2020). Alternatively, the “dedifferentiation” observed in echinoderms may reflect the retention of a more basal, cnidarian-like mechanism, with stem cell dependence arising convergently in other bilaterian groups (Rinkevich et al., 2022). This may explain the vast range of stem cell strategies we see throughout the animal kingdom, most of which are not as extreme as those employed by planarians and acoels (Ferrario et al., 2020; Rinkevich et al., 2022). We speculate that as several bilaterian lineages evolved an increasing number of mature, specialized cell types, trade-offs between somatic function and proliferative potential became more acute, resulting in the increased reliance on a dedicated stem cell class.

Whether we consider blastema cells to arise from dedifferentiated mature cells or from undifferentiated progenitor cells depends to a great degree on how we define these terms. Constructs such as dedifferentiation and stem cells are often very useful, but when we are overly zealous in their use, we risk artificially separating related processes or grouping unrelated processes. Both dedifferentiated and *de novo* progenitor cells may employ homologous genetic circuitry derived from an ancestral regenerative GRNs (Arendt et al., 2016; Srivastava, 2021). With the CoRC concept it is possible that such cells may even be considered the same cell type, as evolutionarily homologous cell types do not necessarily need to arise from the same developmental origins (Arendt et al., 2016; Srivastava, 2021). If we consider regeneration to be a developmental process, the differential employment of stem cells and dedifferentiation in different tissues and species may be analogous to the way in which several mature cell types, such as bone, can arise from multiple embryonic germ layers (Arendt et al., 2016; Srivastava, 2021). Already, efforts have potentially identified an ancestral stem cell regulatory module, which we will discuss more in the next section (Alié et al., 2015; Srivastava, 2021).

### Do conserved gene regulatory networks (GRNs) maintain blastema cell identity?

Given that gene regulatory networks (GRNs) are arguably the best way to establish homology between cell types and regenerative processes (Davidson, 2006; Arendt et al., 2016; Srivastava, 2021), what can our current knowledge of gene expression in the salamander blastema tell us about its evolution? Though several studies have profiled the blastema transcriptome and proteome, the field is only beginning to directly interrogate the functional relationships between specific genetic elements (Rao et al., 2009; 2014; Stewart et al., 2013; Bryant et al., 2017; Gerber et al., 2018; Leigh et al., 2018; Nowoshilow et al., 2018; Sibai et al., 2020; Sousounis et al., 2020). Indeed, the field has only recently identified reliable blastema markers such as *Kazald2* (Bryant et al., 2017), meaning regulatory interactions and mechanistic functions of genes in the salamander blastema must largely be inferred from their roles in more well characterized systems. Salamander blastema cells are enriched for genes associated with stemness in mammals, as well as genes associated with RNA binding and DNA repair, and genes associated with germline maintenance (Chera et al., 2006; Zhu et al., 2012; Stewart et al., 2013; Bryant et al., 2017; Haas and Whited, 2017; Leigh et al., 2018; Nowoshilow et al., 2018).

In mammalian embryos, pluripotency is maintained by a core network of well characterized transcription factors including *Oct4, Klf4, Sox2, Myc* and *Nanog* (Takahashi and Yamanaka, 2006; Liu et al., 2008; Young, 2011). Three of these factors *Myc*, *Klf4*, and *Sox2* are upregulated in the salamander blastema (Maki et al., 2009; Zhu et al., 2012; Stewart et al., 2013; Leigh et al., 2018). Pluripotency-related genes are also expressed in a variety of vertebrate blastemas: lizard blastemas express *cMyc* (Alibardi, 2022b), *Xenopus* blastemas express *cMyc* and *Sox2* (Christen et al., 2010), and lungfish blastemas express *Sox2* and *cMyc* (Nogueira et al., 2016). The widespread employment of these pluripotency genes suggests a conserved roll in vertebrate regenerative processes; however, it is possible that these genes, which are associated with proliferation and “stemness” more generally, were convergently integrated into regeneration programs. A more thorough investigation of the regulatory interactions and elements involved in these processes is needed if we want to properly discern between conservation and convergence (Srivastava, 2021).

Tracing this circuitry beyond the vertebrates is more tenuous (Gold et al., 2014; Srivastava, 2021; Rinkevich et al., 2022). *Oct4, Klf4, Sox2*, and *Nanog* are members of large gene families which diversified dramatically in the deuterostome lineage (Resch et al., 2012; Önal et al., 2012; Gold and Jacobs, 2013; Presnell et al., 2015; Rinkevich et al., 2022). In particular *Oct4* and *Nanog* represent branches of their respective gene families entirely unique to vertebrates (Gold et al., 2014; Scerbo et al., 2014; Sukparangsi et al., 2022). Nonetheless, more distantly-related members of the *Pou* (*Oct4*) family have been found to be upregulated in planarian neoblasts, acoel neoblasts, and cnidarian i-cells, as well as during regeneration in echinoderms and hemichordates (Resch et al., 2012; Önal et al., 2012; Mashanov et al., 2015b; 2015a; Ferrario et al., 2020). This may reflect a regenerative/multipotency role for the ancestral *Pou* family gene in the most ancient metazoans. Challenging this hypothesis, planarian *Pou* genes lack an α-helix domain which is required for pluripotency in vertebrates, and *Pou5/Oct4* paralogs from basal vertebrates (hagfish) fail to maintain a pluripotent state in mouse embryonic stem cells, suggesting the pro-pluripotency function of *Pou5/Oct4* evolved in jawed fish (Gold et al., 2014; Sukparangsi et al., 2022). On the other hand, axolotl and medaka *Pou5/Oct4* and *Pou2* are both competent to induce pluripotency in mouse stem cell models (Tapia et al., 2012). *Pou5*, *Pou2*, and *Pou3* share a common ancestor at the base of bilateria; if this ancestral *Pou2/3/5* gene promoted pluripotency, this role may have been subfunctionalized to different paralogs in different lineages (Gold et al., 2014).

*Myc* and *Sox2* are more deeply conserved and are also found quite widely in invertebrate regeneration. Sox paralogs are upregulated in planarian neoblasts, acoel neoblasts, cnidarian i-cells, and in regenerative echinoderm and tunicates cells (Resch et al., 2012; Önal et al., 2012; Gold and Jacobs, 2013; Mashanov et al., 2015b; 2015a; Reddien, 2018; Rinkevich et al., 2022; Srivastava, 2022). *Myc* is even more widespread; it is expressed during imaginal disc regeneration in *Drosophila*, tunicate regeneration, echinoderm regeneration, cnidarian i-cells, and even sponge archaeocytes, though this gene has notably been lost in acoels and planarians (Gallant, 2013; Gold and Jacobs, 2013; Mashanov et al., 2015b; 2015a; Alié et al., 2015; Rinkevich et al., 2022). *Klf4* has been observed in regeneration in echinoderms (Mashanov et al., 2015a). Other vertebrate pluripotency transcription factor families such as *Gata4/5/6*, *FoxO*, and *Pax* are found in multiple invertebrate stem cells (Brown and Swalla, 2007; Boehm et al., 2012; Somorjai et al., 2012b; Chiodin et al., 2013; Rosner et al., 2013; Alié et al., 2015; Ricci et al., 2016; Somorjai, 2017; Srivastava, 2021; Rinkevich et al., 2022). Does the widespread use of these genes in regenerative processes reflect an ancestral circuit, which has been modified in vertebrates to also include *Oct4* and Nanog, or is this an example of the convergent use of pro-proliferative genes in regenerative processes? Further probing of regulatory factors, binding sites and interactions utilized during regeneration will enable us to discern which elements are truly conserved or derived across the animal kingdom.

Pluripotency is often associated with germline, and salamander blastema cells express many germline-associated genes including the RNA-binding proteins *Piwi*, *Vasa* and *Nanos* (Zhu et al., 2012; Sousa-Victor et al., 2017). Piwi genes in particular are expressed in a wide variety of regenerative cells in invertebrates, including, planarian, acoel, and annelid neoblasts, cnidarian i-cells, as well as regenerative stem cells in ascidians and echinoderms (Reddien et al., 2005b; Seto et al., 2007; Palakodeti et al., 2008; Rinkevich et al., 2010; 2013; Leclère et al., 2012; van Wolfswinkel, 2014; Mashanov V. et al., 2015; Özpolat and Bely, 2016; Lai and Aboobaker, 2018; Kostyuchenko and Kozin, 2021; Hulett et al., 2023). Piwi and other germline markers have also been observed in somatic stem cells of sponges, and ctenophores in addition to the aforementioned groups (Alié et al., 2011; Lai and Aboobaker, 2018; Koutsouveli et al., 2020)**.** The widespread appearance of *Piwi* genes and other germline markers in regenerative and somatic stem cells has led some to propose a conserved GRN, the germline multipotency program, involved with germline maintenance, pluripotency, and regeneration throughout the animal kingdom (Lai and Aboobaker, 2018; Srivastava, 2021).

One potentially very ancient feature of the blastema transcriptome is the general upregulation of RNA binding proteins (RNBPs). Several RNBPs including *cirbp*, *fus*, *roa1*, *safb1*, and *hnrnpd* are upregulated in the axolotl blastema (Bryant et al., 2017). Comparisons between sponge archaeocytes, planarian neoblasts, and hydrozoan i-cells suggest that RNBPs were a major component of their inferred ancestral stem cell regulatory program (Alié et al., 2015). Interestingly several blastema-enriched RNBP homologs are found in sponge archaeocytes, including *CIRBP*, *SAFB1*, and members of the hnRNP family (Alié et al., 2015; Bryant et al., 2017). While these genes may represent an ancient, conserved link between blastema cells and basal metazoan stem cell programs, it is important to caution that these specific genes are not appreciably upregulated in planarian neoblasts, and the most promising candidates for an ancestral stem cell program, such as members of the DDX (*Vasa-*related), family do not appear to be upregulated in the axolotl blastema (Alié et al., 2015; Bryant et al., 2017). As with other examples of shared gene usage in distantly related lineages, untangling convergence and conservation is complicated, requiring a thorough characterization of regulatory relationships (Srivastava, 2021). Importantly, several uncharacterized genes are upregulated in both the salamander blastema and other regenerative models (Alié et al., 2015; Bryant et al., 2017; Rinkevich et al., 2022). The comparative analysis of these genes may eventually reveal important, ancient components of the regenerative circuitry which have been lost in mammals.

## The evolutionary origins of the vertebrate blastema within the greater context of metazoan regeneration

Robust regenerative abilities are found in all basal metazoan groups (Bely and Nyberg, 2010; Tanaka and Reddien, 2011; Slack, 2017; Ricci and Srivastava, 2018), suggesting an early, and most likely shared origin for regeneration throughout the animal kingdom. This regenerative ability likely evolved in parallel to other processes necessary for multicellularity, such as development, growth, wound healing, and reproduction (Bely and Nyberg, 2010; Slack, 2017). Indeed, when we look at the most basal metazoans, we see that the lines between these processes are somewhat blurred (Bely and Nyberg, 2010; Gold and Jacobs, 2013). Many sponges and cnidarians redeploy developmental processes throughout their lifecycle: symmetry breaking and patterning are redeployed in the adult to enforce the appropriate spacing of repeating structures during growth and reproduction (Lengfeld et al., 2009; Watanabe et al., 2014; Soubigou et al., 2020)**.** In particular, Wnt signaling, FGF signaling, and BMP/TGF-β signaling are all employed during regeneration and development in sponges and cnidarians (Gold and Jacobs, 2013; Maddaluno et al., 2017; Slack, 2017; Soubigou et al., 2020; Tursch and Holstein, 2023)**,** and all have significant roles in the axolotl blastema (McCusker et al., 2015; Vincent et al., 2020)**.** These components also have deeply conserved roles in early embryonic development (Slack, 2017; Zinski et al., 2018), and are possibly the most frequently reoccurring components in regenerative systems throughout the animal kingdom (Maddaluno et al., 2017; Slack, 2017)**.**

Within Bilateria, commonalities between planarian and acoel regeneration strongly suggest that the last common ancestor of Bilateria regenerated by a similar mechanism; both employ body wide, totipotent neoblast stem cells during whole-body regeneration (Reddien and Sánchez Alvarado, 2004; Srivastava et al., 2014; Raz et al., 2017). As with cnidarians, these cells are directed by axial gradients of developmental positional control genes, including *Wnts* and *Bmps*, which are expressed throughout the animal’s lifetime (Reddien and Sánchez Alvarado, 2004; Srivastava et al., 2014; Raz et al., 2017; Slack, 2017; Srivastava, 2022). Arguably, the main similarity between axolotl limb regeneration and this proposed ancient bilateral mechanism is the activation of and migration of scattered stem cells to the site of injury during blastema formation (Kragl et al., 2009; Choi et al., 2017; Flowers et al., 2017; Raz et al., 2017; Gerber et al., 2018; Leigh et al., 2018). Interestingly, in both planarians and acoels, neoblasts across the entire body start to proliferate, *in-situ*, shortly after injury, increasing in number before migration (Raz et al., 2017). The proliferative activation of distant stem cells shortly after dramatic injury is seen in many distantly related organisms, including the axolotl, and even in nonregenerative species such as mice, hinting that whole-body stem cell activation may be a remnant of this ancient mechanism (Rodgers et al., 2014; Johnson et al., 2018; Reddien, 2018; Srivastava, 2022; Payzin-Dogru et al., 2023). Perhaps, like planarian neoblasts, globally activated axolotl stem cells are primed to migrate towards the injury site and contribute to the nascent blastema but only those situated close enough to the injury site receive migration and/or blastema-specification signals. Another possibility is that more distant cells are also physically impeded by extracellular matrix, while local matrix is deconstructed in response to amputation. However, further study into this phenomenon is required to address these possibilities.

### Where then does the salamander blastema sit within the greater context of metazoan regeneration?

Slack proposes that an ancient whole-body body regenerative mechanism is conserved across basal metazoans, but that salamander limb regeneration is probably derived (Slack, 2017). This argument rests on the observation that basal metazoans retain body-wide, embryonic axial patterning gradients as adults; these gradients facilitate regeneration in these groups, but have been lost in vertebrates and most bilaterian lineages (Slack, 2017). *While this is one interpretation, we present an alternate hypothesis: salamander limb regeneration is ultimately derived from ancient whole body regenerative mechanisms but is restricted and limited by the developmental and physiological demands of anatomically sophisticated vertebrates.* In this model the axolotl blastema is not an evolutionary novelty, but a vestige of earlier whole-body regenerative mechanisms. Slack argues that because molecules such as Wnts and BMPs are repurposed several times in vertebrate development, the ancestral whole body regenerative mechanism must have been lost in the adult, and limb regeneration must have been reacquired (Slack, 2017). It is possible, however, that vertebrates lost whole body regeneration for other reasons, with limb regeneration being a remnant of this ancient ability. Some hypothesize that vertebrate paired appendages arose through a reactivation of the developmental program that patterns the anterior posterior axis (Shubin et al., 1997)**.** Supporting this hypothesis, the hox genes that pattern the proximal distal axis of the developing and regenerating vertebrate limb are related to, and ordered in the same way as the hox genes which pattern the embryonic anterior-posterior axis (Shubin et al., 1997). Moreover, *Wnts* are expressed distally in the developmental limb-bud, potentially reflecting their role in posterior specification, and BMPs specify ventral tissue, as they do during axial patterning (Shubin et al., 1997; Robert, 2007; Lovely et al., 2022). If axial developmental programs were repurposed for vertebrate limb development, perhaps whole-body regenerative mechanisms were similarly repurposed towards the limb. Of course, this is highly speculative. Whether vertebrate limb regeneration is a vestige of ancestral whole-body regeneration, or an evolutionary novelty depends on whether “the entire ancestral line of animals has had a similar regenerative ability” (Slack, 2017). Recent evidence supports this ancestral continuity: tunicates have extensive regenerative abilities, cephalochordates regenerate the tail through a mechanism at least superficially similar to axolotl regeneration (Ferrario et al., 2020), larval jawless fish such as lampreys can regenerate their tails (Bayramov et al., 2018), sharks have recently been shown to regenerate fins (Lu et al., 2013; Alibardi, 2019; 2022a; Borucinska et al., 2020; Marconi et al., 2020; Womersley et al., 2021), and of course there are many examples of appendage regeneration throughout the bony fish (Yoshinari and Kawakami, 2011; Nogueira et al., 2016; Darnet et al., 2019).

While the relationship between the axolotl blastema and basal metazoan regeneration remains unresolved, we can compare vertebrate blastemas with relative confidence: several features of the salamander limb blastema—Wnt/FGF/BMP signaling, the expression of pluripotency markers, the contribution of fibroblasts, and nerve dependence, are shared by the bony fish fin blastema, as well as the less regenerative lizard tail blastema, and mouse digit tip blastema (Gemberling et al., 2013; McCusker et al., 2015; Nogueira et al., 2016; Payzin-Dogru and Whited, 2018; Darnet et al., 2019; Alibardi, 2022b; Johnson and Lehoczky, 2022). If the salamander blastema represents a vestige of a more flexible ancestral regenerative mechanism, mouse digit tip regeneration may itself represent a vestige of a more salamander-like ancestral amniote regenerative mechanism. Our current understanding of blastema evolution is constrained by lack of diverse model systems. If digit tip regeneration is vestigial, it should be widespread amongst the amniotes, but this trait remains unexamined beyond a handful of placental mammal species. Likewise, sharks, lampreys, and cephalochordates all regenerate fins through mechanisms that superficially resemble the axolotl blastema, but the molecular and cellular circuitry underlying these processes remains largely uncharacterized (Somorjai et al., 2012b; Womersley et al., 2021; Li et al., 2023). As the field explores regeneration in less-characterized species, with an expanding arsenal of molecular and genetic tools, the relationships between well-established regenerative models, such as the mouse digit tip, and the axolotl limb will be more clearly defined. A thorough understanding of blastema evolution will both sate our biological curiosity and facilitate the selection of appropriate models for human regenerative therapies.
